# Supplementary material for: Cytokines Differently Define the Immunomodulation of Mesenchymal Stem Cells from the Periodontal Ligament
Source: Cells. 2020 May 14;9(5):1222. doi: 10.3390/cells9051222 (PMC7290931; doi:10.3390/cells9051222)
Supplement: Supplementary file 1 [file cells-09-01222-s001.pdf]

cells MDPI

Supplementary Information

Article

Cytokines Differently Define the Immunomodulation of Mesenchymal Stem Cells from the Periodontal Ligament

Christian Behm [1,2], Alice Blufstein [1], Johannes Gahn [1], Michael Nemec [2], Andreas Moritz [1], Xiaohui Rausch-Fan [1] and Oleh Andrukhov [1,*]

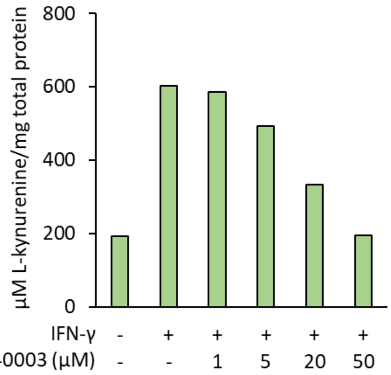

Figure S1. Effect of different PF-06840003 (IDO-1 inhibitor) concentrations on the IDO-1 enzymatic activity. Primary hPDLSCs were treated with 100 ng/ml IFN-γ in the absence or presence of PF-06840003 (1 µM – 50 µM). Unstimulated hPDLSCs served as control. IDO-1 enzymatic activity was measured by quantifying L-kynurenine concentration (µM) in the conditioned media, normalized to the total protein amounts in mg. All data are presented as mean value ± S.E.M. and were received from one experiment with cells isolated from one individual.

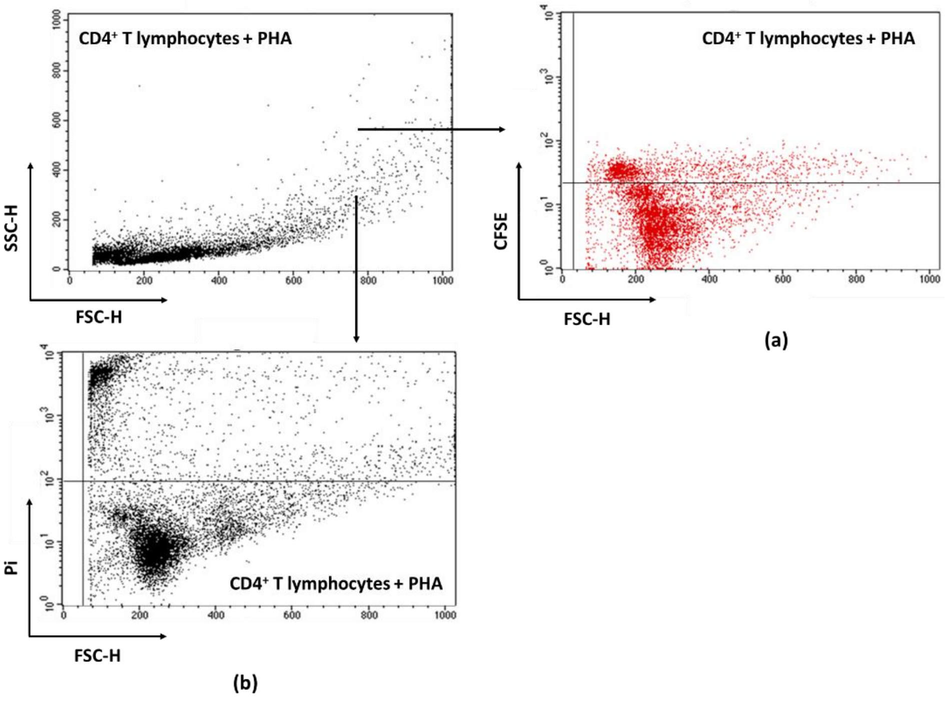

Figure S2. Representative dot plots which outline the gating / analysis strategy for CFSE proliferation (a) and Pi apoptosis (b) assays. From these dot plots the % of at least once divided $CD4^+$ T lymphocytes and the % of Pi positive $CD4^+$ T lymphocytes were calculated.

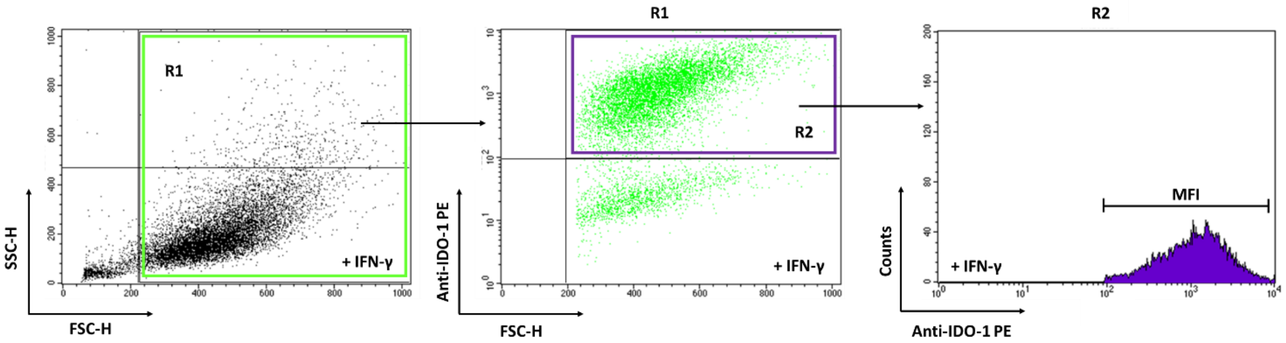

Figure S3. Representative dot plots and one-parameter histogram which outline the gating / analysis strategy for calculating the % of IDO-1 positive hPDLSCs and the corresponding m.f.i.

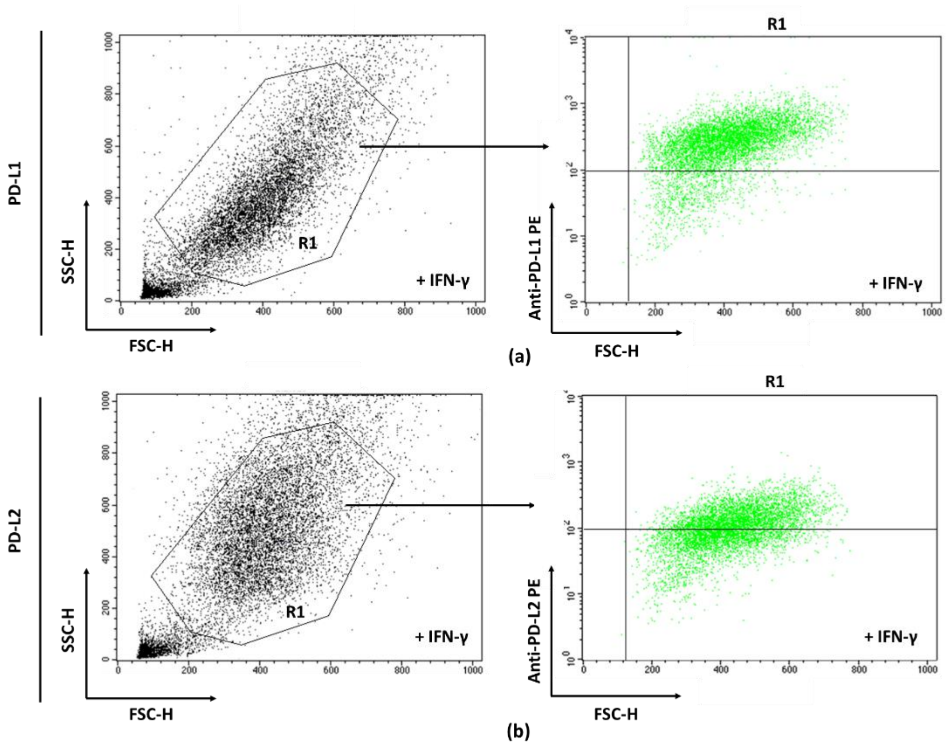

Figure S4. Representative dot plots with outline the gating / analysis strategy for calculating the % of PD-L1 (a) or PD-L2 (b) positive hPDLSCs.

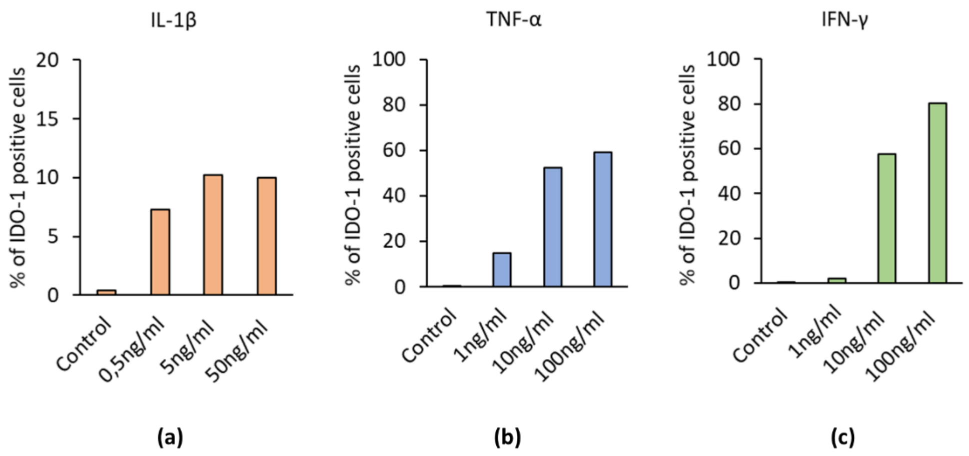

Figure S1. Effect of different IL-1β, TNF-α and IFN-γ concentrations on the percentage of IDO-1 positive hPDLSCs. Primary hPDLSCs were treated with different (a) IL-1β (0.5ng/ml – 50ng/ml), (b) TNF-α (1ng/ml – 100ng/ml) and (c) IFN-γ (1ng/ml – 100ng/ml) concentrations. Unstimulated hPDLSCs served as control. 48 hours after treatment, IDO-1 protein levels were quantified by intracellular immunostaining followed by flow cytometry analysis, determining the percentage of IDO-1 positive hPDLSCs. All data are presented as mean value ± S.E.M. and were received from one experiment with cells isolated from one individual.
